# The yin and yang of amyloid aggregation

**DOI:** 10.4155/fso.15.40

**Published:** 2015-09-01

**Authors:** S Fabio Falsone

**Affiliations:** 1 Institute of Pharmaceutical Sciences, University of Graz, Schubertstr 1, 8010 Graz, Austria

**Keywords:** amyloid fibers, heat shock protein, HSP, molecular chaperones, prion-like

## Abstract

Intra- and extra-cellular amyloid protein fibers are traditionally coupled to a series of devastating and incurable neurodegenerative disorders. Since the discovery of physiologically useful amyloids, our attention has been shifting from pure pathology to function, as amyloid aggregation seems to constitute a basis for the functional and dynamic assembly of biological structures. The following article summarizes how the cell profits from such an unconventional high-risk aggregation at the rim of physiologic utility and pathologic catastrophe.

Proteins are fundamental molecular constituents of biological life, and nature has elaborated robust and evolutionary refined strategies assuring their functionality and efficiency. The implementation of precise, spatially and temporally defined cellular tasks requires a perfect coordination of a protein's activity, localization and turnover.

A major implication for counteracting a protein's functional failure is to guarantee the preservation of its physical integrity, which is given by the 3D fold of the polypeptide chain. Structurally destabilizing processes such as disease-related alterations of the immediate physiological environment or genetically caused amino acid substitutions can severely compromise the function of a protein. As even a partial loss of the polypeptide's original structure can initiate a fatal aggregation process, the cell has adapted to deal with the permanent peril of misfolding-derived protein toxicity. Safety mechanisms comprise the control of a polypeptide's structural quality, the rescue of damaged proteins, and the complete removal of irremediably damaged polypeptides, protein aggregates, or organelles. Consequently, a malfunction is frequently associated with the occurrence of severe protein misfolding disorders.

## The yin: pathology

Amyloid aggregation is a principal manifestation of high conformational variance. Amyloid fiber polymerization is a self-templating process, whereby one protein switches from a singular native fold into an alternative, β-sheet rich amyloid precursor conformation. This process triggers an analogous conversion of homologous polypeptides in an autocatalytic mode. Such a dramatic conformational reshaping implies at least partial unfolding of a polypeptide, which poses undoubtedly a severe threat to the cell, as it goes hand in hand with functional damage. It is thus not surprising that our actual understanding is historically linked to a series of debilitating and prevalently neurologic diseases characterized by the occurrence of clumps of insoluble amyloid aggregates in different brain regions.

In spite of their unique histological hallmarks, amyloid inclusions from different diseases are composed of functionally unrelated proteins sharing very little structural characteristics. What we know so far from *in vitro* studies is that amyloidogenic proteins all have in common a remarkable extent of conformational freedom, which can vary from delimited regions of low structural complexity to total inherent disorder, thus allowing for substantial conformational reorganization. An extreme representative is α-Synuclein (aSyn), the major protein constituent of Lewy bodies found in brain tissue of individuals affected by synucleinopathies. Isolated aSyn is stable as a monomer constitutively devoid of any structure, but it can natively oligomerize into a soluble, transiently folded alpha-helical tetramer [1], as well as into a beta-sheet shaped polymer when incorporated into insoluble amyloid fibers [2]. While these fibrillar aggregates have long been regarded as the pathogenic agents of diverse amyloid diseases, it is now emerging that molecular pathogens consist of toxic folding intermediates [3] somewhere between native and amyloid conformation, whereas the mature amyloid fibers might constitute an 'ultima ratio' to irreversibly neutralize proteotoxic species. However, the kinetic and thermodynamic heterogeneity of these intermediates impedes a precise correlation of structure to toxicity. Indeed, the amyloid folding landscape is dynamically populated by a highly polymorphic and perturbable variety of aggregates differing in size, shape and stability. This considerably complicates a functional and structural taxonomy, and the systematic isolation and characterization of stable conformational intermediates is generally challenging. Yet, the identification of a unique conformational epitope located on the surface of soluble aggregates from different completely unrelated disease-associated proteins accounts for the existence of a unifying aggregation mechanism [4]. This suggests that the pathogenic agents of different amyloid diseases might share similar structural peculiarities and might accumulate via a similar proteotoxic folding deviation.

Despite the lack of accurate structural details, the proteotoxicity of alternatively folded amyloidogenic proteins is likely to be linked to functional impairment resulting from gain or loss of the original protein function. Some of the current models propose that these species might become toxic by physically affecting membrane integrity, by altering protein quality maintenance, by an impaired mitochondrial function, or by deviated protein-protein interactions. The most singular feature of these deteriorated species, however, is to propagate between cells in a self-perpetuating mode, whereby toxic aggregates can cross intercellular barriers and act as seeds to template the conversion of native protein molecules into a pathogenic conformer. While originally observed for prion disorders (transmissible spongiphorm encephalopaties), this non-Mendelian form of infection is apparently intrinsic to amyloid diseases, as it has been described also for key proteins of synucleinopathies, tauopathies and amyotrophic lateral sclerosis, suggesting that all these diseases similarly propagate in a prion-like fashion [5–7].

A specific amyloid converting endotrophic trigger has not been discovered so far, and although the etiology of some disorders can sometimes be linked to genetic mutations, amyloid aggregation is fundamentally characterized by a substantial sensitivity towards a repertoire of remodeling factors such as environmental pathogens (e.g., pesticides), metals, or reactive oxygen species. This contributes to explain the prevalently idiopathic nature of amyloid disorders over relatively less frequent familial cases.

The mechanisms of cell-to-cell transmission are still unclear, but there are indications for an active transport of infectious particles via exosomal pathways [8]. Moreover, a recent study [9] shows that proteotoxic amyloid aggregates can bind directly to the glycosaminoglycan heparan sulfate, a heavily sulfated oligosaccharide component of certain proteoglycans. The latter decorate the extracellular matrix on the surface of cells and usually act as regulators of inflammatory cell migration. This interaction triggers the pinocytotic internalization of the amyloid aggregate, which can consequently unleash its disastrous power within the infected cell. Intriguingly, the same mechanism of infection can be observed for aggregates deriving from the three different amyloidogenic proteins Tau, aSyn and Prp, in further support for a unifying molecular basis of symptomatically diverse amyloid diseases.

The progressive course of amyloid-associated neurodegeneration led to the intriguing hypothesis of a self-sustained directional propagation of infectious amyloid particles along interconnected neuronal pathways. Such a theory rises from the assumption that amyloid pathologies can originate far from the CNS, and consecutively move to the brain owing to the template-driven self-replicating ability of the infectious agent [10]. Parkinson's disease (PD), for instance, is a condition traditionally associated with the occurrence of Lewy bodies in degenerated dopaminergic motor neurons of the brain. The appearance of identical amyloid inclusions in the enteric system led to postulate an onset of the disease in the gut preceding the development of visible motor symptoms by decades [11]. These observations suggest a peripheral outbreak of the pathology before reaching the CNS, and the release of aSyn into the extracellular space of enteric neurons might contribute to the spreading of aggregates along neurons that extend up to the CNS. This might also account for the development of nonmotoric PD symptoms, such as frequently observed digestive disorders, preceding the classical motoric handicaps.

## The yang: physiology

In spite of the impending hazards described above, the cell seems to rationally exploit amyloid aggregation for physiological purposes. As several proteins from widespread organisms indeed polymerize into amyloids with well-defined functions and without any immediate toxicity, it appears that evolution tolerates in some measure the survival of potentially harmful polypeptide variants. Even more bizarre, the proteome of some organisms, such as of the social amoeba *Dictyostelium discoideum*, displays an intriguingly high amount of low complexity regions formed of repetitive amino acid stretches of variable length, in particular glutamine residues [12]. These are prone to aggregate in form of amyloid after a threshold length has been exceeded, and in humans, the variability of polyglutamine repeats directly correlates with the severity of a series of debilitating disorders, such as Huntington's disease or spinal and bulbar muscular atrophy.

All these issues imply that cells must pursue a robust hedge management in order to functionally benefit from aggregation, which leads to ask about the intrinsic motivation of playing for high stakes at the verge of lethality.

One possible answer comes from mammalian secretory granules, where a set of peptide hormones is present in form of amyloid-like aggregates capable of releasing monomeric units under determinate conditions [13]. The rationale seems to be the requirement of a stable storage facility for the rapid release of active hormone upon acute demand. As the characteristic cross-β amyloid geometry allows for a tight polymer stacking, inactive hormone can be packed up to very high local concentrations, which might obviate the need of a repeated *de novo* synthesis without affecting the immediate availability of the substance. The further confinement of hormone polymers within a coating membrane provides a means for cells to store amyloid under relatively innocuous conditions.

One further impressive answer comes from yeast, an organism that has learned to proficiently couple amyloid transmission to phenotypic variation. Yeast possesses a set of proteins that share self-templating properties typical of those observed for amyloids in humans and are reminiscent of mammalian prion proteins, except for the frequent lack of toxicity of the infectious conformer. One cardinal feature of yeast is to support prion propagation from mother to daughter cell, a property that establishes a paradigmatic link between yeast prion inheritance and non-Mendelian phenotypic variation [14]. Investigations on the yeast prion prototype Sup35 revealed that a structural rearrangement from a native nonamyloidogenic [PSI] into a self-perpetuating amyloidogenic [PSI+] conformation was essential for inheritance. In striking contrast to mammalian prions, [PSI+] is not stringently toxic, and its stabilization is in some cases accompanied by a remarkable acquisition of novel cellular phenotypes. The molecular basis of phenotypic variation can be ascribed to a functional loss when soluble Sup35, which is a release factor that controls the fidelity of ribosome translation termination, converts into unsoluble amyloid. Generally, this protein's impairment promotes the release of cryptic gene variants upon epigenetic pressure. Some of them lead to the exposition of phenotypes with an increased chance of evolutionary survival. At the end, yeast bargains the preservation of a phenotypic status quo with selective adaptation in an unparalleled utilitaristic scenario. Therefore, the perspective of benefitting from new phenotypic traits pays off for a possible failure of actual protein homeostasis. This provides a formidable representation of how protein folding couples environmental stress to phenotypic variation.

The aquatic snail *Aplysia californica* also provides a remarkable example of how an organism can recruit amyloid fibers for physiological operations. Amyloid fibers of the RNA-binding protein CPEB are still functional, in that they retain the ability to actively discriminate polyadenylation sequences on 3-UTRs of mRNA [15]. The superior physical stability of the amyloid state over the native monomer of CPEB apparently turns to an advantage, as polyadenylation of multiple RNAs can be efficiently coordinated in a spatially localized manner, which seems to be essential for the long-term mnemonic activity of this snail.

With the support of knowledge-based algorithms which can be specifically trained to distinguish the physical composition of particular amino acid patterns, it has been possible to define prion-like attributes of polypeptides in terms of sequence similarities. Prion-like regions are frequently characterized by stretches of low structural complexity containing an unusually high amount of glutamine and asparagine residues (Q/N-rich regions) [16]. The subsequent experimental verification of a huge set of predicted yeast prion candidates led to the discovery of previously unsuspected proteins capable of phenotypic variation based on a monomer to amyloid transition typical of ‘genuine’ prions. In alignment with experimental results from case-by-case studies, prion-like consensus sequences possess modular features, as they are sufficient to autonomously sustain amyloid spreading when heterologously fused to proteins or when interchanged between organisms. Amyloid aggregates from the computationally identified prion-like translational repressor Pum from *Drosophila melanogaster*, for instance, acquire heritable traits when recombinantly produced in yeast [17]. Similarly, the Q/N-rich region from human stress granule component TIA-1 can be replaced with one from unrelated yeast Sup35 and vice versa, without affecting the amyloidogenic properties of both proteins [18].

As inferred from global surveys, a significantly high number of proteins with predicted or validated prion-like properties localizes within RNP bodies, which are organelles that broadly regulate RNA metabolism and turnover. RNP bodies, which are composed mainly of ribonucleoprotein particles, operate as RNA processing machines that accomplish different specialized tasks either constitutively (such as Cajal Bodies, gems, or P-Bodies) or upon definite request (e.g., stress granules). In clear-cut contrast to secretory granules, RNP bodies are not equipped with any coating membrane, and it is likely that these organelles exploit prion-like aggregation to gain a physical integrity. Stress granules, for instance, are a type of RNP body representatives that accumulate rapidly in the cytosol upon acute stress. They retain untranslated mRNA molecules as long as homeostasis has not been restored, and disassemble afterwards. TIA-1, which is the principal protein component of stress granules, exploits its prion-like characteristics to initiate the reversible assembly of these particular RNP bodies. These can no longer assemble when the prion-like region of TIA-1 is deleted, whereby this ability is restored upon substitution with a heterologous prion-like region [18].

There are increasing indications for an amyloid-like nature of RNP granules, such as the identification of fibrous core structures within P-bodies [19], which are organelles that supervise the degradation of untranslated mRNA in close collaboration with stress granules. It has further been observed, that highly concentrated solutions of purified prion-like granule components readily condensate *in vitro* into amyloid-like hydrogels [20] with morphologic and functional patterns typical of RNA granules. Their ability to reversibly incorporate a considerable number of additional granule-specific mRNA and protein components from cellular extracts emulates the remarkable dynamics of stress granules and in general of RNA bodies, which profit from the absence of a membrane for a vivid and relatively uncomplicated interchange of molecules with the immediate environment.

While of advantage when acute stimuli require a rapid (dis)assembly or reorganization of stress granules, the lack of a confining membrane bears the hazard of a cell's excessive exposure to latently toxic aggregates. Indeed, stress granules act as nuclei for the heterotypic aggregation of a whole series of structurally metastable constituents of RNA regulating machineries with documented or suspected implications in neurodegenerative disorders [20]. With respect to these proteins, the term ‘supersaturated’ has been introduced to define polypeptides whose cellular concentrations are high in relation to their solubility [21]. These proteins are generally inclined to aggregation, with an equilibrium that can be easily tilted to unsoluble. As such, they precipitate upon an excess of aberrantly stabilized beta-strand rich polypeptides [22] or small organic molecules which are likely to mimic a catalytic surface for amyloid aggregation [20]. Thus, the exaggerated or uncontrollable persistence of stress granules, and more generally of amyloid in the cell, can come along with the precipitation and consequent failure of entire protein circuits.

## Yin & yang: strategies to control amyloid equilibrium

To overcome these inherent dangers, the cell has developed multilevel backup strategies to keep protein aggregation reversible. These include: keeping the abundance of aggregation-prone proteins constitutively low; raising their concentration above a critical aggregation threshold only upon stringent necessity and in a temporally and spatially restricted mode; and neutralizing irreversibly aggregated proteins in case of serious challenge [23].

While the abundance of aggregation-prone proteins can be kept minimal by both a remarkably low stability of their coding mRNAs and their mediocre translation efficiency, the transition from monomer to aggregate for functional purposes imposes the manipulation of a protein's physical properties. This can occur either via post-translational modifications of aggregation-prone regions (e.g., frequently observed phosphorylation) [24], or by providing extrinsic scaffolds that organize the assembly of monomeric units. In this regard, it is intriguing to suppose that RNA polymers might initiate the generation of RNP bodies by cooperatively modulating the assembly of many prion-like constituents [25]. We further like to suggest that the recently discovered protein class MOAG-4/SERF [26,27] might represent a dedicated scaffold for amyloid aggregation, as it displays the unique ability to distinguish between amyloid and nonamyloid aggregation without being actively incorporated into amyloid polymers, with a consequent significant accumulation of soluble amyloid intermediates.

With nonspecific aggregation prevailing over functional aggregation during severe challenge, cells must mobilize superior aggregation modifying mechanisms to efficiently neutralize the toxicity of misfolded or heavily damaged polypeptides. For this purpose, organisms have established an ingeniously tunable, omnipresent and compensatory platform, which builds upon the interplay between the protein class of molecular chaperones and protease machineries. The former supervises the folding integrity of polypeptides, while the latter removes those which fail folding quality criteria. Together, they orchestrate a powerful decisional network that minimizes the accumulation of proteotoxic species by means of qualitative selection and disposal [28], embracing virtually the entire amyloid folding landscape. Different molecular chaperone representatives can control early (the heat shock proteins hsp70 and hsp90) as well as late (hsp104) amyloidogenesis. While hsp70 and hsp90 seem to partition toxic from nontoxic polypeptides, hsp104 is a disaggregase that solubilizes high molecular size aggregates and mature amyloid fibers. Remarkably, hsp104 is absent in metazoans, and given that mature fibers are most likely nontoxic, it can be speculated that in higher eukaryotes there is no immediate requirement for their complete dissolution.

In close conjunction, two major proteolytic pathways account for both basal protein turnover and the elimination of polypeptides which have been sorted for proteolytic clearance. These are the ubiquitin-proteasome system (UPS) and autophagy, which can be subdivided into chaperone mediated autophagy (CMA), macroautophagy (MA) and microautophagy. While the proteasome and CMA operate as single throughput proteases in direct collaboration with molecular chaperones, MA and microautophagy can remove bulk cellular components up to entire organelles.

The partially redundant overlap of multiple chaperone – and proteolytic pathways – has a compensatory advantage, since one malfunctioning complex can be usually backed by the mutual activation of at least another complex, keeping the toxic burden innocuously low. Such a fail-safe strategy is peculiar to intransigently counteract the risks arising from conformational heterogeneity typical of amyloidogenic proteins. By these means, it is possible to direct aggregates to the most qualified clearance system, but also to hand them over for alternative disposal. As an example, high molecular size aggregates accumulating as a consequence of malfunctioning CMA or UPS can be passed to macroautophagosomes for bulk elimination [29]. However, the amount of energy paid for the synergistic upkeep of these very large and sophisticated protein complexes is conspicuously high. Consequently, serious energetic disequilibria as observed in neurodegenerative disorders (e.g., mitochondrial damage) [30], can severely and globally compromise the fine-tuned interplay and stability of these machineries. This might explain the significant amount of folding/degradation components incorporated within amyloid inclusions of affected brains.

## Conclusion & future perspective

The concept of amyloid aggregation is shifting from purely pathologic to functional. Although we are now starting to understand that physiologic amyloids can exist as a result of a highly regulated and stringently balanced process, the next big challenge is to pinpoint the causes that overthrow this balance from beneficial to toxic. This step is fundamental to overcome obstacles in the identification of suitable pharmacological targets and disease markers posed by the heterogeneous and highly variable nature of amyloid aggregation.

**Executive summary**

The term ‘amyloid’ describes a particular class of unbranched protein aggregates with typical β-cross structure.
Amyloid aggregation is traditionally associated with neurodegenerative disorders.
Organisms exploit amyloid polymerization for functional purposes.
The cell possesses multiple backup mechanisms to profit from functional aggregation while minimizing the risk of unspecific aggregation.
The derangement from functional to pathologic aggregation is probably due to the failure of sorting and degradation mechanisms.
